# Louhi 2014: Special issue on health text mining and information analysis

**DOI:** 10.1186/1472-6947-15-S2-S1

**Published:** 2015-06-15

**Authors:** Sumithra Velupillai, Martin Duneld, Aron Henriksson, Maria Kvist, Maria Skeppstedt, Hercules Dalianis

**Affiliations:** 1 Department of Computer and System Sciences, (DSV), Stockholm University, P. O. Box 7003, 164 07 Kista, Stockholm, Sweden

## Introduction

Mining and automating analysis of information from health documents holds great potential for improving health care in many aspects. Health documents include text sources such as medical records, scientific publications, and user-generated texts in e.g. social media. Research in the area of health text mining has grown and matured in recent years. Research on texts written in English is dominating, but in the last ten years research on other languages has evolved, including work on French, Spanish, Swedish, Danish, Finnish, German, Chinese and Japanese health documents. Initially, scientific biomedical articles were the main data source, after which research on clinical notes, blogs and patient forums, etc. has also increased. Moreover, the number of practical applications has increased [1], including tools for de-identification of Protected Health Information (PHI) in clinical text [2], automatic diagnosis code assignment [3], clinical decision support [4], detection of adverse drug events [5], as well as other adverse hospital events such as detection of hospital acquired infections [6], extraction of structured information from pathology reports [7], text simplification of clinical text for patient use [8], as well as pre-processing pipelines and information extraction in tools such as cTAKES [9].

Working with sensitive data such as medical records requires considerable efforts and considerations in terms of ensuring individual privacy, which makes access to such data an obstacle. Through the organization of shared tasks such as i2b2 (https://www.i2b2.org/NLP/, accessed 2 Nov. 2014), ShARe/CLEF eHealth (http://clefehealth2014.dcu.ie/, accessed 2 Nov. 2014) and SemEval (http://alt.qcri.org/semeval2014/task7/, accessed 2 Nov. 2014), English datasets have been made more easily available for research groups, which has been a crucial step towards strengthening method development and ensuring comparability as well as reproducibility. For other languages, very few such resources are currently available. Similarly, a very important resource for performing research in the health text domain is the availability of terminologies and ontologies. SNOMED CT is a global comprehensive clinical terminology available in English, French, Danish, Dutch, Spanish and Swedish. Extending this and similar terminologies to new languages is a necessity.

This supplement aims at contributing to the research community in the area of information analysis in the health domain by presenting work describing approaches, solutions and progress in the challenges identified above. Specifically, it is of importance that research on languages other than English is presented, as well as novel data resources. Research in natural language processing and text mining of health documents is increasing and has extended to several new languages such as German, Finnish, Spanish and Basque, as well as to new application areas. We believe that the evolvement and progress in this area will increase the efficiency and quality of health care in the near future.

## Summary of contributions

In this special issue, we present research articles following the 5th Louhi workshop held at the EACL conference in Gothenburg, Sweden, in April 2014 [10]. The Louhi workshop series was initiated in 2008 in Turku, Finland, and has since resulted in the publication of international state-of-the-art research in the health text mining domain [11-14].

All accepted and published contributions to the 5th Louhi workshop were invited to submit substantially extended and revised studies to this supplement, and submitted contributions were peer-reviewed by three program committee members, with at least one of them also having been a reviewer for the original workshop contribution. From the 17 accepted workshop papers published in the EACL proceedings, eight extended submissions were received for this special issue, and five were accepted after the peer-review process. We thank all authors and reviewers for their hard work and engagement!

In order of appearance, each paper is briefly summarized below.

Electronic health records (EHR) contain information about a patient's status and treatment in the form of sequential documentation - clinical notes. From admission to discharge, these notes form care episodes. EHRs are used throughout the health care sector primarily for clinical purposes, but also for secondary purposes such as decision support and research. To support evidence-based medicine, the undertaken task is to, given a care episode, retrieve the most similar care episodes among the records so that a patient's situation and possible outcome can be compared to earlier patients. Moen et al. present several methods for care episode retrieval, based on textual similarity, where similarity is measured through domain-specific modelling of the distributional semantics of words in the free text of the EHR. Models include variants of random indexing and the semantic neural network model word2vec. Two novel methods are presented that utilize the ICD-10 codes attached to care episodes to better induce domain-specificity in the semantic model, and an experimental evaluation of care episode retrieval that circumvents the lack of human judgements regarding episode relevance is presented. Results suggest that several of the proposed methods outperform a traditional search engine on the retrieval task.

Alnazzawi et al. describe annotation of a corpus aimed at encoding detailed phenotypic information. Mentions of concepts relating to congestive heart failure were annotated (e.g. causes, risk factors and signs & symptoms), as well as several types of relationships between them. Identified concepts were also mapped to UMLS. To make systems that are to be developed on the corpus more robust to different text types, discharge summaries as well as full-text scientific articles were selected for annotation. Three different methods for named entity recognition of mentioned concepts were also evaluated; dictionary-based, rule-based as well as different types of machine learning methods. The rule-based methods produced the best results, but the best performing machine learning model, for which manual rule construction is not required, achieved competitive performance.

Kreuzthaler & Schulz describe work on detecting sentence boundaries and abbreviations in German clinical text by developing supervised classifiers (support vector machines) for each task. Text snippets were annotated by two researchers - where the role of a period character was classified as an abbreviation and/or sentence marker. A number of features were defined for the two tasks, including statistical corpus features and different scaling combinations on these, as well as rule-based and dictionary-dependent features, and an extensive analysis on feature impact and combinations was performed. This is the first study on this task applied on German clinical narratives in this way.

In the study by Perez-de-Viñaspre & Oronoz, the process of designing and developing a partial implementation of semi-automatic translation of medical terms in the English version of SNOMED CT to the Basque language is described. The method depends on four phases to complete the process, and in this article the first two phases are described in detail and results of initial experiments are presented. They have with the initial phases succeeded to translate a fifth of the disorder terms into Basque and a tenth of the terms for body structures. The general interest of this article lies in that this study describes a method for less resourced languages to acquire vocabularies.

The article by Segura-Bedmar et al. addresses the nascent notion of exploiting user-generated data to support pharmacovigilance - an area that suffers from gross underreporting of adverse drug events - and, importantly so, targets a language other than English: Spanish. The development of a corpus, comprising user comments from a health networking site and annotated with drugs and their effects, is described. The corpus is then used to evaluate a system that aims to automate the extraction of drugs, as well as their indications and side-effects. The system is based on the distant supervision paradigm, i.e., using a database, in this case of drugs and their indications and side-effects, to label examples for supervised machine learning. A system was constructed by employing shallow features, the recall of which improved by ten percent, albeit at the expense of precision, compared to a co-occurrence and knowledge-based system.

## Programme committee

Antti Airola, University of Turku, Turku, Finland

Beáta Megyesi, Uppsala University, Uppsala, Sweden

David Martinez, University of Melbourne and MedWhat.com

Dimitris Kokkinakis, University of Gothenburg, Gothenburg, Sweden

Filip Ginter, University of Turku, Turku, Finland

Gintaré Grigonyté, Stockholm University, Stockholm, Sweden

Jon D. Patrick, Health Language Laboratories, Australia

Jong C. Park, KAIST Computer Science, Korea

Mats Wirén, Stockholm University, Stockholm, Sweden

Pierre Zweigenbaum, LIMSI, Computer Sciences Laboratory for Mechanics and Engineering Sciences, France

Sabine Bergler, Concordia University, Canada

Sampo Pyysalo, University of Tokyo, Japan

Sanna Salanterä, University of Turku, Finland

Sophia Ananiadou, University of Manchester, UK

Stefan Schulz, Graz General Hospital and University Clinics, Austria

Tapio Salakoski, University of Turku, Finland

Thomas Brox Røst, Norwegian University of Science and Technology, Norway

## Competing interests

The authors declare that they have no competing interests.

## Declarations

Publication costs for this article were funded by the corresponding author's institution: Department of Computer and System Sciences, (DSV), Stockholm University.

This article has been published as part of *BMC Medical Informatics and Decision Making *Volume 15 Supplement 2, 2015: Proceedings of Louhi 2014: The Fifth International Workshop on Health Text Mining and Information Analysis. The full contents of the supplement are available online at http://www.biomedcentral.com/bmcmedinformdecismak/supplements/15/S2.
